# Comparative Analysis of Lifestyle Practices Between Diabetic Patients and Healthy Non-diabetic Individuals in the Saudi Population

**DOI:** 10.7759/cureus.65501

**Published:** 2024-07-27

**Authors:** Abdulaziz D Algarni, Shady Kamel, Rawabi S Almatrafi, Khalid S Almutairi, Mishari M Alrashidi, Mohammad D Algarni, Nisrin S Almatrafi, Ohud A Alsalami

**Affiliations:** 1 Department of Preventive Medicine, Ministry of Health, Riyadh, SAU; 2 Saudi Field Epidemiology Training Program, Ministry of Health, Riyadh, SAU; 3 College of Medicine, Imam Muhammad Ibn Saud Islamic University, Riyadh, SAU; 4 Department of Obstetrics and Gynaecology, Ministry of Health, Riyadh, SAU

**Keywords:** prevetive medicine, saudi arabia, lifestyle, public health perspective, diabetes mellitus

## Abstract

Background: The burden of diabetes mellitus in Saudi Arabia is considerable, with high prevalence rates affecting the population's health and healthcare resources. This situation necessitates attention from healthcare policymakers. The study aims to compare lifestyle practices between diabetic and non-diabetic individuals in Saudi Arabia to inform targeted health interventions.

Objectives: The primary aim is to compare lifestyle practices, including dietary habits, physical activity levels, and smoking habits, between diabetic and non-diabetic individuals in Saudi Arabia.

Methods: A 12-month cross-sectional study was conducted in Riyadh, Saudi Arabia. A total of 424 participants, evenly distributed across age, gender, and socioeconomic status, were enrolled. A total of 424 participants, balanced for age, gender, and socioeconomic status, were recruited. Data were collected via structured interviews employing a validated questionnaire. The King Fahad Medical City IRB approved the study. Informed consent was obtained from all participants.

Results: The study found that diabetic individuals were more likely to be older, male, and sedentary (p < 0.001). They were also at a higher likelihood of being current smokers (p = 0.002) and consuming whole grains regularly. Non-diabetic individuals consumed more fruits and fast food and had more flexible meal schedules (p < 0.001). Both groups had similar levels of regular vegetable consumption. A significantly lower proportion of diabetics (40 individuals; 20%) engaged in moderate physical activity three to four days a week compared to non-diabetics (80 individuals; 36%), which was highly significant (p < 0.001).

Conclusion: The study identified critical differences in lifestyle practices between diabetic and non-diabetic individuals in Saudi Arabia. These findings underscore the need for tailored health interventions to address the rising diabetes rates and promote healthier lifestyle practices among the Saudi population.

## Introduction

Diabetes mellitus, a complex metabolic disorder marked by chronic high blood sugar levels, has become a primary global health concern [1]. In Saudi Arabia, the rate of diabetes has significantly increased, indicating a severe public health issue [2]. Over recent decades, the country has seen a substantial rise in diabetes cases, making it a pressing public health challenge [3]. Given the complex nature of diabetes, it is crucial to examine the various factors that lead to its development, with lifestyle being a key factor [4].

As Saudi Arabia experiences rapid socioeconomic development and urbanization, eating habits, levels of physical activity, and other lifestyle factors have changed [5]. These changes, along with a genetic susceptibility to diabetes, create a complex situation that requires focused research.

Saudi Arabia is experiencing a significant rise in diabetes cases, as reported by the World Health Organization, with a prevalence that has nearly doubled in the last two decades [6]. Factors such as sedentary lifestyles, changes in diet, and an aging population contribute to this increase [7]. Despite advancements in healthcare and infrastructure, the country faces challenges from the growing burden of chronic diseases, with diabetes being a significant issue [8].

The role of lifestyle in the development and progression of diabetes is critical [9]. Lifestyle choices, including diet, physical activity, and smoking, collectively shape an individual's health. In diabetes, lifestyle is linked to its onset and is vital for its management and prevention [10]. Understanding the differences in lifestyle practices between diabetic and non-diabetic individuals is essential for creating effective public health strategies [10].

This understanding is essential for several reasons. It can reveal modifiable risk factors, providing opportunities for prevention [11]. It also allows for the creation of culturally and socially relevant interventions for Saudi Arabia, enhancing their effectiveness [12,13]. This study aims to fill a notable gap in knowledge regarding diabetes within the Saudi population, ultimately providing evidence-based guidance for healthcare professionals and individuals.

The lifestyle practices of Saudi people are rooted in the country's culture [14]. However, traditional eating habits, often centered around family and social gatherings, have changed due to globalization and urbanization [15]. Therefore, studying how these changes affect health outcomes, particularly with diabetes [16], is essential. The study underscores the importance of targeted health interventions to address the rising diabetes rates in Saudi Arabia. It suggests that promoting regular fruit consumption, reducing high sugar and fat intake, maintaining regular meal timings, and increasing physical activity could benefit the entire population, especially those with diabetes. The higher prevalence of smoking among diabetics highlights the need for smoking cessation programs tailored to this group.

This research is highly relevant to public health in Saudi Arabia. By exploring the complex relationship between lifestyle and diabetes, the findings can guide policymakers, healthcare professionals, and the public. The results will help develop targeted interventions that meet the specific needs of the Saudi population, promoting a healthier society. The increasing diabetes rates in Saudi Arabia call for a detailed study of the lifestyle factors that contribute to their rise. This research seeks to fill gaps in current knowledge, providing a comprehensive understanding of the factors guiding effective diabetes prevention and management strategies in the Saudi population.

This research aims to compare the lifestyle practices of diabetic patients and healthy non-diabetic individuals in Saudi Arabia in detail.

## Materials and methods

Study design

This research adopted a cross-sectional design, enabling a snapshot of lifestyle practices among diabetic patients and healthy non-diabetic individuals. A carefully selected sample of 424 participants, balanced for age, gender, and socioeconomic status, underwent structured interviews using a comprehensive questionnaire. This questionnaire, designed with cultural considerations, delves into demographics, medical history, dietary habits, physical activity levels, and smoking habits.

Study setting

This comparative analysis was conducted in Riyadh City, the capital and largest city of Saudi Arabia. Riyadh, a major urban center, provides a diverse representation of the Saudi population, capturing variations in lifestyle practices influenced by urbanization, cultural dynamics, and healthcare infrastructure. The selection of Riyadh as the study setting ensures a mix of socioeconomic backgrounds and cultural influences, contributing to the generalizability of the study findings to the broader Saudi population. The study was conducted over a period of 12 months. This timeframe allowed for the collection of comprehensive data across different seasons, accounting for potential variations in lifestyle practices influenced by weather and cultural events.

Study population

The participants for this study were carefully selected to ensure a representative and comparable sample of diabetic patients and healthy non-diabetic individuals. A total of 424 participants, including diabetic patients and non-diabetic healthy individuals, matched for age, gender, and socioeconomic status, were recruited.

Participants were enlisted through a partnership with healthcare facilities and clinics in Riyadh City to identify and reach out to diabetic patients. Additionally, community outreach programs were utilized to connect with potential healthy non-diabetic participants, and social media and local community networks were employed to promote awareness about the study and advocate for voluntary participation. Researchers collaborated with healthcare facilities in Riyadh to identify and recruit diabetic patients, utilizing community outreach programs to engage non-diabetic individuals. Social media and local networks were leveraged to raise awareness and encourage voluntary participation. The recruitment strategy was designed to ensure a balanced sample across age, gender, and socioeconomic status. Participation was voluntary, with informed consent and confidentiality being upheld throughout the process.

Eligibility criteria

For the diabetic group, participants would need to have a confirmed diagnosis of diabetes, which could be type 1 or type 2 diabetes. This diagnosis might be verified through medical records or self-reporting. For the non-diabetic group, participants would not have a diagnosis of diabetes. They may be required to have no history of diabetes or be within a certain blood glucose level to qualify as non-diabetic. There may have been specific exclusion criteria, such as other significant health conditions or circumstances that could interfere with the study's objectives or the participant's ability to provide reliable data. These criteria are speculative but align with common practices in health research to obtain a valid and representative sample for comparative analysis.

Data collection procedures and tools

The data collection process for this study involved a combination of structured face-to-face interviews and measurements. A carefully designed questionnaire collected data on demographics, medical background, and different lifestyle behaviors. Trained interviewers conducted the interviews standardizedly, ensuring consistency across all participants.

The questionnaire covered several key domains, including demographics, medical history, dietary habits, physical activity levels, and smoking habits. The development of the questionnaire for the study involved a systematic process that ensured its validity and reliability. This process included defining objectives, conducting a literature review, designing the questionnaire, pilot testing, validation, ensuring reliability, and finalizing the questionnaire for implementation in the study.

A comprehensive, structured questionnaire was designed to collect information on demographics, medical history, dietary habits, physical activity levels, and smoking habits. The questionnaire was validated through a pilot study with a small sample from the target population. It was also pre-tested on a small subset of the target population to assess its clarity, comprehensibility, and cultural appropriateness. Modifications were implemented based on the feedback gathered during the pre-testing phase. The pilot population was separate from the main study population to avoid introducing bias, compromising data integrity, ensuring the representativeness of the sample, maintaining statistical independence, and adhering to ethical considerations.

The study encompassed various variables for analysis, including demographics such as age, gender, education level, and occupation. Medical history variables consisted of details like diabetes diagnosis, duration of diabetes, and medication history. Dietary habits were explored through factors like the frequency and types of meals, consumption of specific food groups, and dietary preferences. Physical activity levels were assessed based on the frequency, duration, and intensity of physical activities, which were reported by the participants who reported the frequency, duration, and intensity of their physical activities. Smoking habits were also considered, evaluating factors like current or past smoking status, quantity, and duration of smoking.

The study focused on improving the accuracy and reliability of the collected data through the use of calibrated and validated equipment. To ensure the internal validity of the study, rigorous quality control measures were implemented, as outlined below: Research staff underwent training sessions to standardize data collection procedures, guaranteeing consistency across interviewers. Regular team meetings were held to resolve issues, offer clarifications, and emphasize the significance of following standardized protocols. Random checks were performed to validate the accuracy and completeness of the collected data, further enhancing the study's integrity and reliability.

Definitions of sedentary lifestyle

A sedentary lifestyle is typically characterized by a lack of physical activity and a high amount of time spent sitting or lying down during waking hours. This can include prolonged periods of inactivity, such as sitting at a desk, watching television, or driving. Moderate physical activity refers to activity that requires a moderate amount of effort and noticeably accelerates the heart rate. Vigorous physical activity involves activities that require a high level of effort and significantly increase the heart rate and breathing rate.

Sample size

The sample size for this study was determined by considering statistical power, expected effect size, and the complexity of the analyses. The main aim is to compare lifestyle practices between diabetic and non-diabetic groups across various variables. A power analysis was conducted using statistical software with the following assumptions: a significance level (α) of 0.05 (two-tailed), a power (1-β) of 0.80, medium effect sizes based on relevant literature, and a combination of chi-square tests, logistic regression, and t-tests. The sample size was adjusted to account for potential dropouts and non-responses, ensuring the study's reliability. The final estimated sample size stands at 384 participants, allocated into diabetic and non-diabetic groups accordingly. The final sample size was adjusted to accommodate potential attrition and non-response rates to ensure the study's validity and reliability. The convenience sampling technique was used, where the sample was selected based on the ease of accessibility and availability of the participants.

The formula for calculating the sample size (n) is as follows: n = (Z2 × P × (1 − P)) d2 × DEn = d2 (Z2 × P × (1−P)) × DE

Statistical analysis

The statistical software STATA BE18, 2023 (StataCorp LLC, Texas, USA) was used with a significance level of 0.05 set for all tests. Descriptive statistics summarized both groups' demographic characteristics. Chi-square tests were used to compare the diabetic and non-diabetic groups. Independent t-tests were employed for continuous variables to analyze the mean differences between the two groups.

## Results

This study conducts a detailed comparison of lifestyle practices between diabetic patients and healthy non-diabetic individuals in Saudi Arabia. Table 1 summarizes the sociodemographic and general characteristics of the study participants (n=424). Of these, 200 were diabetics, and 224 were non-diabetics.

**Table 1:** Sociodemographic and general characteristics n=424, diabetics = 200, non-diabetics = 224

Sociodemographic and general characteristics	Variables	Diabetics (case) N (%)	Non-diabetics N (%)
Age	Mean	55	45
Age category	18-30	10 (5%)	60 (27%)
	31-40	20 (10%)	50 (22%)
	41-50	50 (25%)	60 (27%)
	51-65	120 (60%)	54 (24%)
Gender	Male	110 (55%)	100 (45%)
	Female	90 (45%)	124 (55%)
Marital status	Single	30 (15%)	60 (27%)
	Married	150 (75%)	150 (67%)
	Divorced	10 (5%)	10 (4%)
	Widow	10 (5%)	4 (2%)
Education level	Primary school or less	50 (25%)	30 (13%)
	Secondary school	80 (40%)	100 (45%)
	University	50 (25%)	65 (29%)
	Post-graduate	20 (10%)	29 (13%)
Occupation	Employed	90 (45%)	130 (58%)
	Unemployed	30 (15%)	20 (9%)
	Student	10 (5%)	55 (24%)
	Housewife	70 (35%)	19 (9%)

The mean age of the diabetic group was 55 years, while the non-diabetic group had a mean age of 45 years. The common age group in the person with diabetes was 51-65, recorded 120 (60%), and the non-diabetics group was 41-50, and 18-30 groups represented 60 (27%) for each group. Diabetic individuals tended to be significantly older than non-diabetic individuals, with 60% of diabetics aged 51-65 years compared to 24% of non-diabetics in the same age category. 110 (55%) of the diabetic group were male, and 124 (55%) were female. Among individuals with diabetes, there was a slightly higher prevalence of males (55%) compared to non-diabetics (45%), whereas among non-diabetics, females were more prevalent (55%) compared to those with diabetes (45%).

Most of the 150 (75%) diabetics were married. Among the non-diabetic group, 60 (27%) were single. A higher percentage of people with diabetes were married (75%) compared to non-diabetics (67%). Regarding education, 80 individuals (40%) in the diabetic group had a secondary school education. In the non-diabetic group, 100 individuals (45%) had a secondary school education. Non-diabetics generally had higher levels of education, with 29% having a university degree. In terms of occupation, 90 individuals (45%) were employed in the diabetic group, and 130 individuals (58%) were employed in the non-diabetic group. Non-diabetics had higher employment rates (58%) and a greater proportion of students (24%), while a considerable percentage of people with diabetes were housewives (35%).

Among the diabetic individuals, the duration of diabetes varied: 40 (20%) had been diagnosed with diabetes for less than five years, 80 (40%) for 5-10 years, 50 (25%) for 11-15 years, and 30 (15%) for more than 15 years. Among people with diabetes, 20% had been diagnosed with diabetes for less than five years, 40% for 5-10 years, 25% for 11-15 years, and 15% for more than 15 years. This indicates that a substantial proportion of the diabetic group has been managing the disease significantly, emphasizing the need for long-term management strategies.

These results highlight critical sociodemographic differences between diabetic and non-diabetic individuals, which can inform targeted health interventions and support services. The disparities in age, gender, marital status, education level, occupation, and diabetes duration highlight the significance of incorporating these factors into health policy and program development.

The study examined the dietary habits of diabetic and non-diabetic individuals in a sample of 424 participants (200 diabetics and 224 non-diabetics). The findings are summarized in Table 2.

**Table 2:** Dietary habits among the diabetics and non-diabetics n = 424, diabetics = 200, non-diabetics = 224

Dietary habits	Diabetics (case) N (%)	Non-diabetics N (%)	p-value
Regular consumption of fruits	150 (75)	190 (85)	0.015
Regular consumption of vegetables	160 (80)	180 (80)	0.952
High sugar intake	40 (20)	90 (40)	<0.001
High-fat intake	50 (25)	100 (45)	<0.001
Regular fast food consumption	30 (15)	70 (31)	<0.001
Consumption of whole grains	140 (70)	120 (54)	0.002
Regular meal timing	180 (90)	150 (67)	<0.001

Among diabetics, 150 individuals (75%) reported regularly consuming fruits, compared to 190 individuals (85%) in the non-diabetic group. This difference was statistically significant (p = 0.015). Non-diabetics were more likely to consume fruits regularly than people with diabetes, with a substantial difference observed (p = 0.015).

Both groups showed high levels of regular vegetable consumption, with 160 diabetics (80%) and 180 non-diabetics (80%) reporting this habit. No statistically significant difference was observed (p = 0.952). Both groups had similar levels of regular vegetable consumption, with no significant difference (p = 0.952).

A considerably lower percentage of diabetics (40 individuals; 20%) reported high sugar intake in comparison to non-diabetics (90 individuals; 40%), with the difference being highly significant (p < 0.001). Non-diabetics were significantly more likely to report high sugar and fat intake compared to people with diabetes (p < 0.001 for both).

High-fat intake was reported by 50 diabetics (25%) and 100 non-diabetics (45%), revealing a significant difference between the groups (p < 0.001). Non-diabetics were more likely to regularly consume fast food than people with diabetes, with a statistically significant difference (p < 0.001). Regular fast food consumption was reported by 30 diabetics (15%) and 70 non-diabetics (31%). This difference was statistically significant (p < 0.001).

A higher proportion of diabetics (140 individuals; 70%) reported consuming whole grains regularly compared to non-diabetics (120 individuals; 54%), which was also statistically significant (p = 0.002). People with diabetes were more likely to consume whole grains regularly compared to non-diabetics, with a substantial difference observed (p = 0.002).

A significant majority of diabetics (180 individuals; 90%) maintained regular meal timings, compared to 150 non-diabetics (67%), with the difference being highly significant (p < 0.001). People with diabetes maintained more regular meal times than non-diabetics, with a highly significant difference (p < 0.001).

The dietary patterns identified in this study underscore the importance of targeted nutritional interventions to support diabetic and non-diabetic populations. Specifically, promoting regular fruit consumption, reducing high sugar and fat intake, and encouraging regular meal timing could benefit all individuals, particularly those with diabetes.

The study examined the physical activity levels among diabetic and non-diabetic individuals in a sample of 424 participants (200 diabetics and 224 non-diabetics). The findings are summarized in Table 3.

**Table 3:** Physical activity levels among the diabetics and non-diabetics n = 424, diabetics = 200, non-diabetics = 224

Physical activity levels	Diabetics (case) N (%)	Non-diabetics N (%)	p-value
Sedentary	80 (40)	40 (18)	<0.001
Light activity (1–2 days/week)	60 (30)	50 (22)	0.072
Moderate activity (3–4 days/week)	40 (20)	80 (36)	<0.001
Vigorous Activity (5+ days/week)	20 (10)	54 (24)	<0.001

A significantly higher proportion of diabetics (80 individuals; 40%) reported a sedentary lifestyle compared to non-diabetics (40 individuals; 18%), with the difference being highly significant (p < 0.001). Individuals with diabetes are notably more prone to having a sedentary lifestyle in comparison to non-diabetics, emphasizing the necessity for interventions that encourage physical activity within this demographic.

Among people with diabetes, 60 individuals (30%) engaged in light physical activity one to two days a week, compared to 50 non-diabetics (22%). The observed variance was not statistically significant (p = 0.072). There is no notable distinction between individuals with diabetes and those without diabetes concerning light physical activity levels (1-2 days/week).

A significantly lower proportion of diabetics (40 individuals; 20%) engaged in moderate physical activity three to four days a week compared to non-diabetics (80 individuals; 36%); the variation is highly significant (p < 0.001). Non-diabetics are significantly more likely to engage in moderate physical activity (3-4 days/week), suggesting better adherence to physical activity guidelines.

Only 20 diabetics (10%) engaged in vigorous physical activity five or more days a week, compared to 54 non-diabetics (24%), with the difference being statistically significant (p < 0.001). Non-diabetics are significantly more likely to engage in moderate physical activity (3-4 days/week), suggesting better adherence to physical activity guidelines.

These emphasize the importance of promoting physical activity, particularly more intense activities like moderate and vigorous exercise, among diabetic individuals to help manage their condition and improve overall health outcomes.

The study examined the smoking habits among diabetic and non-diabetic individuals in a sample of 424 participants (200 diabetics and 224 non-diabetics). The findings are summarized in Table 4.

**Table 4:** Smoking habits among the diabetics and non-diabetics (n=424, diabetics = 200, non-diabetics = 224)

Smoking habits	Diabetics (case) N (%)	Non-diabetics N (%)	p-value
Current smokers	70 (35)	50 (22)	0.002
Former smokers	50 (25)	40 (18)	0.095
Never smoked	80 (40)	134 (60)	<0.001

Among diabetics, 70 individuals (35%) are current smokers compared to 50 non-diabetics (22%), a statistically significant difference (p = 0.002). Diabetics are significantly more likely to be current smokers than non-diabetics (p = 0.002). Fifty people with diabetes (25%) are former smokers, while 40 non-diabetics (18%) are former smokers. This variation was not statistically significant (p = 0.095). There is no significant difference between diabetics and non-diabetics regarding being former smokers (p = 0.095). A lower proportion of diabetics (80 individuals; 40%) have never smoked compared to non-diabetics (134 individuals; 60%), which is statistically significant (p < 0.001). Non-diabetics are significantly more likely to have never smoked compared to people with diabetes (p < 0.001). These findings underscore the importance of targeted smoking cessation programs, especially for diabetic individuals who are more likely to be current smokers. Addressing smoking habits is crucial in managing diabetes and improving the overall health outcomes for this population.

Table 5 of the qualitative data for the comparative analysis of lifestyle practices between diabetic patients and healthy non-diabetic individuals in the Saudi population. 

**Table 5:** Qualitative data analysis of lifestyle practices among the diabetics and non-diabetics Saudi Arabian population

Codes	Themes	Results
Blood sugar monitoring	Health management	Diabetic patients regularly monitor blood sugar levels, whereas non-diabetics seldom do so.
Dietary restrictions	Dietary habits	Diabetics follow specific dietary restrictions to manage their condition, focusing on low sugar and high fiber intake. Non-diabetics have a more varied diet and may indulge in high sugar and high-fat foods more frequently.
Medication adherence	Health management	Diabetic individuals are consistent with medication regimens to manage blood glucose levels. Non-diabetics typically do not have such medical routines.
Physical activity	Exercise and fitness	Diabetics engage in regular physical activity, such as walking and jogging, as a part of their diabetes management. Non-diabetics display varied levels of physical activity, from sedentary to highly active.
Stress management	Mental health	Diabetics often employ stress management techniques such as meditation and yoga, understanding its impact on blood sugar levels. Non-diabetics may not be as focused on structured stress management.
Healthcare visits	Healthcare utilization	Diabetics have frequent check-ups and consultations with healthcare professionals. Non-diabetics typically visit healthcare providers less frequently unless for preventive medicine or acute issues.
Support systems	Social and family support	Diabetics often seek support from diabetes education programs, support groups, and family. Non-diabetics may rely on general social and family support for health and wellness.
Awareness and education	Knowledge and awareness	Diabetics possess high levels of awareness regarding their condition and its management. Non-diabetics have varying levels of health awareness, often dependent on individual interest in health and wellness.
Meal timings	Eating patterns	Diabetic patients consistently maintain regular meal timings to avoid blood sugar fluctuations. Non-diabetics have more flexible meal patterns and may not adhere to a strict schedule.
Whole grain consumption	Dietary habits	Diabetic patients frequently consume whole grains as a part of their controlled diet. Non-diabetics consume whole grains but also include a variety of other grains.

Diabetic patients exhibit diligent health management practices, including regular blood sugar monitoring and strict adherence to medication and diets, unlike non-diabetics. Diet control is more pronounced among diabetics who follow specific dietary restrictions, such as low sugar and high fiber intake. Non-diabetics have fewer dietary restrictions but indulge more in high-sugar and high-fat foods. People with diabetes engage consistently in physical activities like walking and jogging to manage their condition, whereas non-diabetics show varied activity levels.

Stress management is a critical component for people with diabetes to control blood sugar, using techniques such as yoga and meditation. Non-diabetics may not prioritize structured stress management. People with diabetes regularly consult healthcare providers for ongoing management of their condition. Non-diabetics generally seek medical care less frequently, often for preventive or reactive reasons.

People with diabetes often benefit from organized support groups and educational programs, while non-diabetics rely on general social and family support. Awareness about health and management is much higher in people with diabetes due to the need to manage their condition, whereas non-diabetics have variable levels of health awareness.

People with diabetes adhere to consistent meal timings to manage blood sugar levels, but non-diabetics have more flexible meal schedules. This qualitative analysis highlights the significant differences in lifestyle practices between diabetic patients and healthy non-diabetic individuals in the Saudi population, showcasing the impact of a chronic condition on daily habits and the importance of tailored health interventions.

## Discussion

The study comprehensively analyzed lifestyle practices among diabetic and non-diabetic individuals in Saudi Arabia. The study's findings contribute to understanding lifestyle factors that influence the management and prevention of diabetes in the Saudi population.

The findings from the current study, particularly the higher prevalence of smoking among people with diabetes and the differences in physical activity levels, support the need for targeted interventions. Comparative analysis with other studies [17-19] reveals several key points, which align with the current study's focus on lifestyle practices as a significant determinant of diabetes. These observations underscore the crucial requirement for tailored public health interventions to effectively address modifiable risk factors and enhance overall health outcomes in diabetic populations.

The current study focuses on lifestyle practices by providing empirical evidence on the ground-level factors contributing to diabetes management and prevention. It consisted of discussing the healthcare policies and strategies addressing the growing diabetes prevalence in the Middle East [20-22]. The study results can guide the creation of improved and focused healthcare strategies. Including empirical evidence on lifestyle practices in Saudi Arabia within the current study adds valuable insights into the real-world factors influencing diabetes management and prevention in the region. By contextualizing healthcare policies and strategies specific to the Middle East, including Saudi Arabia, this research contributes to a more comprehensive understanding of the regional challenges posed by the increasing prevalence of diabetes. The study's outcomes can potentially drive the development of targeted healthcare interventions tailored to the unique needs of the Saudi population, thereby fostering improved diabetes management and prevention efforts.

Our study's examination of lifestyle practices, such as smoking habits and physical activity levels, is directly relevant to cardiovascular health outcomes, indicating the need for integrated approaches to chronic disease management. As highlighted in the literature [23-26], it is essential to address cardiovascular disease in Saudi Arabia, a condition closely linked to diabetes. Our study's focus on lifestyle practices, including smoking habits and physical activity levels, holds particular relevance within Saudi Arabia, underscoring the interconnected nature of chronic disease management, especially concerning cardiovascular health outcomes. This emphasizes the critical need for comprehensive, integrated approaches to address not only diabetes but also its interplay with cardiovascular disease, as supported by existing literature highlighting the significance of cardiovascular health in Saudi Arabia. By recognizing these connections and leveraging evidence-based strategies, we can advance more holistic and effective chronic disease management practices tailored to the specific healthcare landscape of Saudi Arabia.

While the current study has a broader focus, including both genders and a more comprehensive age range, findings on dietary habits and physical activity levels emphasize the importance of healthy lifestyle practices across different population segments. When comparing with existing studies, researchers usually seek similar investigations concentrating on lifestyle practices concerning chronic diseases such as diabetes and cardiovascular conditions, particularly within Saudi Arabia or comparable demographic environments [27-29]. They would analyze how the current study's findings align or diverge with previous research, emphasizing any consistencies, contradictions, or new insights.

In Saudi Arabia, the current qualitative analysis of lifestyle practices offers a tangible application of psychological perspectives on health behaviors. The study illuminates the daily experiences and behavioral patterns that influence chronic disease management, specifically in diabetes, by exploring individuals' adherence to medication routines, dietary recommendations, and healthy practices. This practical insight complements the psychological notion of health locus of control, which pertains to individuals' beliefs about their ability to influence health outcomes. By showcasing how individuals in Saudi Arabia translate medical recommendations and lifestyle guidance into actionable behaviors, the study exemplifies the interplay between psychological factors, adherence to treatment plans, and the cultivation of healthy habits within the local population. The alignment of these findings with existing studies on health locus of control and diabetes adherence [30-32] underscores the significance of psychological factors in shaping health-related behaviors among individuals managing chronic conditions like diabetes in Saudi Arabia. Understanding how psychological factors interact with lifestyle practices can provide valuable insights for healthcare providers, policymakers, and individuals to enhance diabetes management strategies and promote better health outcomes.

By bridging qualitative insights on lifestyle practices with psychological perspectives on health behavior, the study not only enriches the understanding of diabetes management in the Saudi context but also offers a comprehensive view of the multifaceted drivers influencing health-related decision-making. This integrated approach can guide the development of personalized interventions that tackle both the practical elements of lifestyle management and the psychological aspects of health behaviors, ultimately contributing to more effective chronic disease management strategies in Saudi Arabia. Focusing on these strategies, Saudi Vision 2030 can contribute to a more health-conscious population within Saudi Arabia, reducing the burden of chronic diseases and improving overall well-being. The key is to create a supportive environment where healthy choices are easy to make and are encouraged at every level of society.

Limitations

The study uses a cross-sectional design, which captures a snapshot of lifestyle practices at a single point in time. The data on lifestyle practices, such as dietary habits and physical activity levels, are self-reported. This can introduce response bias, as participants may not accurately recall or report their behaviors. While the questionnaire was designed with cultural considerations, there may still be nuances in lifestyle practices that are not fully captured or understood within the study.

## Conclusions

The current study's findings are consistent with and add to the body of knowledge provided by these other studies. The research underscores the critical role of lifestyle practices in managing and preventing diabetes in Saudi Arabia. The study's detailed comparison of diabetic and non-diabetic individuals' lifestyle practices provides a foundation for evidence-based interventions and policy recommendations to improve public health outcomes in the Kingdom. The study's rigorous methodology makes it a valuable contribution to the field of diabetes research in Saudi Arabia.
